# Hematological and Biochemical Laboratory Parameters in COVID-19 Patients: A Retrospective Modeling Study of Severity and Mortality Predictors

**DOI:** 10.1155/2023/7753631

**Published:** 2023-11-18

**Authors:** Ghazaleh Alizad, Ali Asghar Ayatollahi, Armin Shariati Samani, Saeed Samadizadeh, Bahman Aghcheli, Abdolhalim Rajabi, Britt Nakstad, Alireza Tahamtan

**Affiliations:** ^1^Department of Immunology, Faculty of Medicine, Golestan University of Medical Sciences, Gorgan, Iran; ^2^Laboratory Sciences Research Center, Golestan University of Medical Sciences, Gorgan, Iran; ^3^School of International, Golestan University of Medical Sciences, Gorgan, Iran; ^4^Department of Microbiology, Faculty of Medicine, Golestan University of Medical Sciences, Gorgan, Iran; ^5^Environmental Health Research Center, Biostatistics & Epidemiology Department, Faculty of Health, Golestan University of Medical Sciences, Gorgan, Iran; ^6^Division of Paediatric and Adolescent Medicine, University of Oslo, Oslo, Norway; ^7^Department of Paediatrics and Adolescent Health, University of Botswana, Gaborone, Botswana; ^8^Infectious Diseases Research Center, Golestan University of Medical Sciences, Gorgan, Iran

## Abstract

**Background:**

It is well known that laboratory markers could help in identifying risk factors of severe illness and predicting outcomes of diseases. Here, we performed a retrospective modeling study of severity and mortality predictors of hematological and biochemical laboratory parameters in Iranian COVID-19 patients.

**Methods:**

Data were obtained retrospectively from medical records of 564 confirmed Iranian COVID-19 cases. According to the disease severity, the patients were categorized into two groups (severe or nonsevere), and based on the outcome of the disease, patients were divided into two groups (recovered or deceased). Demographic and laboratory data were compared between groups, and statistical analyses were performed to define predictors of disease severity and mortality in the patients.

**Results:**

The study identified a panel of hematological and biochemical markers associated with the severe outcome of COVID-19 and constructed different predictive models for severity and mortality. The disease severity and mortality rate were significantly higher in elderly inpatients, whereas gender was not a determining factor of the clinical outcome. Age-adjusted white blood cells (WBC), platelet cells (PLT), neutrophil-to-lymphocyte ratio (NLR), red blood cells (RBC), hemoglobin (HGB), hematocrit (HCT), erythrocyte sedimentation rate (ESR), mean corpuscular hemoglobin (MCHC), blood urea nitrogen (BUN), and creatinine (Cr) also showed high accuracy in predicting severe cases at the time of hospitalization, and logistic regression analysis suggested grouped hematological parameters (age, WBC, NLR, PLT, HGB, and international normalized ratio (INR)) and biochemical markers (age, BUN, and lactate dehydrogenase (LDH)) as the best models of combined laboratory predictors for severity and mortality.

**Conclusion:**

The findings suggest that a panel of several routine laboratory parameters recorded on admission could be helpful for clinicians to predict and evaluate the risk of disease severity and mortality in COVID-19 patients.

## 1. Introduction

Severe acute respiratory syndrome coronavirus-2 (SARS-CoV-2), which is responsible for the 2019 coronavirus disease (COVID-19), has been recognized worldwide and is referred to as the global pandemic of the century [1, 2]. As of January 2023, at the time of writing this article, about 665 million cases with different clinical symptoms, from mild to moderate to severe to critical, have been diagnosed, and more than 6.7 million deaths from COVID-19 have been reported worldwide [3]. Amidst the critical topics discussed during the COVID-19 pandemic is how to provide an early diagnosis and management of patients to control the outbreak efficiently. Early stages of the disease usually exhibit a mild clinical spectrum; thereby, timely detection, accurate screening, and isolation of infected individuals are vital in minimizing the risk of transmission and managing the disease [4].

Among laboratory diagnostic tests, real-time reverse transcription-polymerase chain reaction (rRT-PCR) is currently considered the most popular test for diagnosing COVID-19 patients [5, 6] Nevertheless, due to its unsatisfactory test sensitivity, rRT-PCR is prone to false negative results in low viral load samples, primarily in patients representing mild disease manifestations [4]. False-negative and false-positive results could lead to improper disease management and negative impacts on a pandemic containment program [7]. Several studies have shown that analyzing clinical and laboratory parameters in diseases assists in identifying risk factors of severe illness, predicting outcomes [8], and allocating proper medical resources at all levels of care to discount morbidity and mortality [9, 10]. Furthermore, considering laboratory parameters could be a cost-efficient and rapid diagnostic strategy in the later stages of an outbreak [11].

Laboratory hematological and biochemical markers may help to predict COVID-19 prognosis [12]. Many studies have pinpointed various prognostic markers, including D-dimer, C-reactive protein (CRP), lactate dehydrogenase (LDH), and high-sensitivity cardiac troponin, in serum of COVID-19 patients with poor outcomes [8, 9, 13, 14]. Deep analysis of abnormal levels of such factors and the interface between their functions in body organs and mechanisms of viral infection can provide the basis for first-line diagnosis as an efficient screening tool [9]. Further knowledge requires sufficient sampling from different countries and populations to power the research, advance health informatics, and turn the data into clinically valuable conclusions. In the present study, we assessed multiple laboratory hematological and biochemical markers of COVID-19 patients in Iran.

## 2. Methods

This retrospective case series was performed on data collected from 564 confirmed COVID-19 cases (based on RT-PCR test) admitted to the referral hospital, 5 Azar Hospital, Gorgan, north of Iran, during February and November 2020. A logistic regression was conducted to examine the odds of death based on independent variables, employing a sample size of 546 observations. The analysis achieved 80% power at a significance level of 0.05, enabling the detection of a change in the probability of *Y* = 1 from the baseline value of 0.2 to 0.31034. This change corresponds to an odds ratio of 1.8. The analysis utilized a two-sided Wald test. Demographic (sex and age) and laboratory data (hematological and biochemical findings) were obtained from patients' electronic records and case record forms at admission. The patients were classified into severe or nonsevere groups based on the level of care received, intensive care unit or not. The patients were also classified into recovered or deceased groups based on their disease outcome. All the patients were from Golestan Province and had the same geographical origin. The study was approved by the Research Ethics Commission of Golestan University of Medical Sciences (IR.GOUMS.REC.1401.070).

Statistical analyses were performed using SPSS 22.0 software. Descriptive analysis variables were expressed as median (interquartile range (IQR)), mean ± SD, or number (%) when appropriate. The Kolmogorov-Smirnov test was performed to check the normality of the variables. Demographic and laboratory data were compared between groups (severity and outcome) using Student's *t*-test for normal distribution; otherwise, the Mann–Whitney test was used. Sex was compared using the *χ*^2^ test, and age was shown as mean ± standard deviation.

To include appropriate parameters in the logistic regression model, we first checked the univariate relationships between the parameters and severity and mortality, and then variables were included in the multiple models. Binary logistic regression analysis was also performed to define independent predictors of disease severity and mortality in COVID-19 patients. To remove the effects of potential confounding factors, i.e., the age parameter in the model, we used the enter method with parameters of a *P* value greater than 0.05 removed for the final model with the forward Wald test. The Hosmer-Lemeshow goodness-of-fit test was used to evaluate how well the model fit with data, reflecting the association between predicted and observed risk. A receiver operating characteristic (ROC) curve was utilized for the predictive performance of disease severity and mortality. *P* values less than 0.05 were regarded as statistically significant. Data for the Bonferroni correction analyses are available for each hypothesis separately.

## 3. Results

In this study, among 564 COVID-19 patients admitted to the referral hospital, 5 Azar Hospital, Gorgan, northern Iran, from February to November 2020, 298 cases were men (52.8%), and 266 cases were women (47.2%). The average age of the patients was 55.6 ± 16 years. Among 564 cases, 97 (17.2%) were deceased, and 467 (82.8%) recovered and could be discharged from hospital. One hundred four (18.5%) and 460 (81.5%) were in the severe and nonsevere groups, respectively. In terms of age, the severe and deceased groups were significantly older than the nonsevere and recovered groups. There was no significant relationship concerning sex between the studied groups (Table 1).

As shown in Table 2, the levels of several hematological parameters, namely, WBC, LYM, NEU, NLR, EOS, RBC, HGB, PLT, HCT, MCHC, ESR, INR, and PT, are significantly different in those who died during hospitalization due to COVID-19 disease compared to those who recovered. Further, the levels of WBC, LYM, NEU, NLR, MO, EOS, RBC, HGB, PLT, HCT, MCHC, INR, and PT were different in severely versus nonseverely diseased groups. Some other parameters did not differ between deceased and recovered groups or those who needed intensive care (severe disease) or were not severely affected.

Table 3 shows significant differences in the level of biochemical parameters such as BUN, Cr, Na, K, Ca, P, LDH, AST, ALP, CPK, D-dimer, D-bili, T-bili, UA, and albumin in deceased compared to recovered groups. There were also significant differences in the level of BUN, Cr, Na, K, Ca, P, LDH, ALP, CK-MB, D-dimer, D-bili, T-Bili, CRP, and albumin between severe and nonsevere groups.

The results of multiple logistic regression models for predictive factors of disease severity and mortality are shown in Table 4. All regression models were age-adjusted to avoid confounding effects on the relationship between severity and mortality. Gender did not significantly differ between groups and was not included in the model as a confounder. Based on the Pearson correlation coefficient, NLR was significantly correlated to lymphocyte and neutrophil counts and percentage and was included in the regression analysis.

In the age-adjusted multiple model, parameters WBC, NLR, ESR, INR, PT, creatinine, BUN, Na, K, P, LDH, ALP, CRP, and D-dimer were independent risk factors of mortality. PLT, RBC, HGB, MCHC, HCT, and albumin were independently protective factors for mortality (Table 4). Moreover, WBC, NLR, INR, PT, BUN, creatinine, K, P, LDH, D-dimer, and ALP were all independent risk factors for the severity of COVID-19 disease. In addition, PLT, RBC, HGB, HCT, MCHC, and albumin were independent protective factors for COVID-19 severity (Table 4).

We then assessed the discriminative power of age-adjusted laboratory variables in predicting the risk of disease severity and mortality in COVID-19 patients by calculating the area under the receiver operating characteristic (ROC) curves (AUC). The results are shown in Table 5.

**Figure 1:**
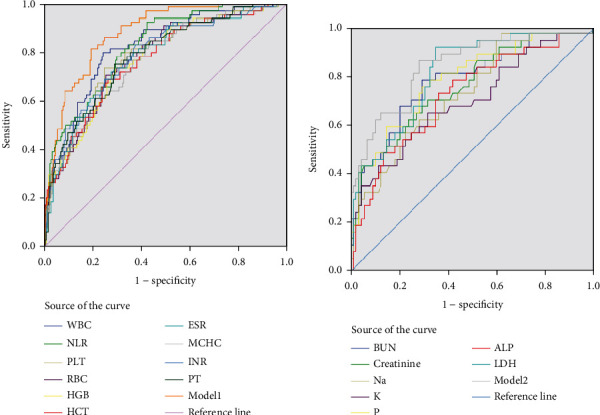
Receiver operating characteristic (ROC) curves for the adjusted laboratory parameters with age and model for prediction of mortality in COVID-19 patients. The analysis of AUCs (area under the curve) for (a) WBC, NLR, PLT, RBC, HGB, HCT, and MCHC and model 2 (b) BUN, creatinine, Na, K, P, LDH, ALP, D-Dimer, CRP, and Albumin.

**Figure 2:**
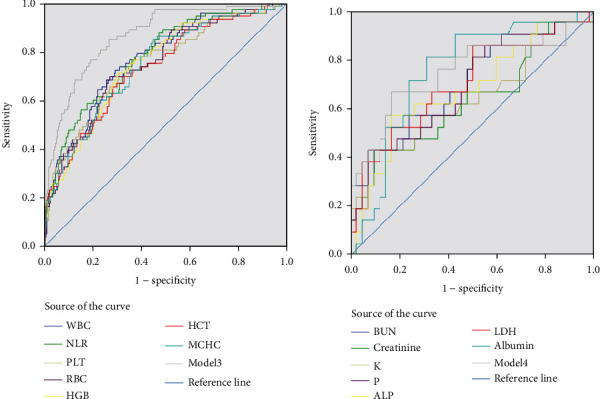
Receiver operating characteristic (ROC) curves for the adjusted laboratory parameters with age and model for prediction of disease severity. The analysis of AUCs (area under the curve) for (a) WBC, NLR, PLT, RBC, HGB, HCT, MCHC, INR, and PT and model 1 (b) BUN, creatinine, K, P, LDH, ALP, D-Dimer, Albumin, and total-bilirubin.

Logistic regression analysis revealed the best models for combined laboratory predictors for severity and mortality. Model 1 contained a combination of five common laboratory predictors and age variables among hematological parameters (AUC 0.87; 95% confidence interval (CI: 0.83-0.91)). Model 2 included a combination of two common laboratory predictors, age and biochemical parameters (AUC 0.85; 95% CI: 0.80-0.91), which showed a better ability to predict mortality than model 1 (Table 5). Logistic regression found model 3 to be the best model for the hematological parameters (AUC 0.86; 95% confidence interval (CI: 0.81-0.90)), whereas model 4 was best for biochemical parameters to predict disease severity (AUC 0.85; 95% confidence interval (CI: 0.80-0.91)) (Figures 1 and 2).

## 4. Discussion

Early identification of patients with a potential to develop severe or critical COVID-19 helps to reduce fatality rates and efficient utilization of limited medical resources [15]. Determining predictive laboratory variables can assist physicians in treating and managing patients in time. Previous studies showed that severe COVID-19 patients had more comorbidities, higher levels of LDH, D-dimer, CRP, leukocytes, and neutrophils, as well as lower levels of albumin, platelet, and lymphocyte counts [16–18]. While these parameters are typically normal at admission, they usually worsen with the course of the disease [19]. Moreover, it is demonstrated that although COVID-19 affects people at any age, more serious consequences may occur among the elderly [20]. In this study, we identified a panel of hematological and biochemical markers associated with the severe outcome of COVID-19 and constructed different predictive models for severity and mortality. Based on the results, the disease severity and mortality rate were significantly higher in elderly inpatients, whereas gender was not a determining factor of the clinical outcome. In this study, age-adjusted WBC, PLT, NLR, RBC, HGB, HCT, ESR, MCHC, BUN, and creatinine also showed high accuracy in predicting severe cases at the time of hospitalization, and logistic regression analysis suggested grouped hematological parameters (age, WBC, NLR, PLT, HGB, and INR) and biochemical markers (age, BUN, and LDH) as the best models of combined laboratory predictors for severity and mortality.

The virus-induced cytokine storm changes the immune cells' homeostasis, resulting in severe outcomes with leukocytosis and neutrophilia and susceptibility to bacterial infections [21]. The systemic inflammation caused by the infection, especially in severe and critical cases, leads to an accelerated migration of lymphocytes from the peripheral blood to the lungs, which could be a reason for subsequent lymphopenia [22]. Results of previous studies are consistent with ours in that patients deceased from COVID-19 showed higher numbers of leukocytes and neutrophils but lower platelets, lymphocytes, and eosinophils [23, 24]. Neutrophil counts were significantly higher in the studied patients, and neutrophilia is shown to be associated with poor outcomes such as acute respiratory distress syndrome, intensive care, and mortality. Lymphocytes, the cells that immune responses to viral infections primarily rely on, have been reported with low numbers in severe cases of COVID-19. Accordingly, levels of neutrophil to lymphocyte ratio could be a useful index for early diagnosis of severe COVID-19 [25]. In the study, in agreement with another study [12], we observed high levels of NLR correlate with disease severity and mortality with an odds ratio of 1.1. NLR also had the best single-parameter differential diagnostic effectiveness (NLR AUC = 0.80), suggesting it is a promising predictive index. Altogether, while leukocytosis, lymphopenia, and neutrophilia were found as important predictive risk indicators for disease severity and mortality, eosinophils and monocytes did not represent a strong correlation with COVID-19 outcomes, according to the present study.

Our study aligns with another study [26], demonstrating a significant decrease in RBC, HGB, HCT, and MCHC values among critically ill and deceased patients, but not in healthier patients. Although HGB level varies in different studies [26–28], the lower levels might be due to the intense inflammation disrupting progenitor cell and erythrocyte functions. Viral inflammation also affects other hematological parameters. Several studies have reported significant alterations in specific coagulation indices, such as the level of D-dimer, prothrombin time, INR, and the number of platelets [29]. A considerably higher incidence of disseminated intravascular coagulation (DIC) has been reported among COVID-19 patients who died from the severe disease compared with those who survived (71.4% vs. 0.6%) [30]. Coagulation cascade activation contributes to higher mortality and is possibly related to the changes in platelet production and destruction in viral infections. This results in coagulation imbalance and microthrombosis in the lungs and other organs [8, 31]. The resulting thrombocytopenia (platelets less than 150 × 109/L) seems to be an important indicator of COVID-19 severity and fatal outcomes [31]. In this study, the platelet levels were significantly lower in the severe and deceased groups compared to the nonsevere and recovered groups, although the average platelet level was generally within normal ranges. Data regarding PTT in COVID-19 patients varied in previous studies [30, 32], which may be an effect of therapies and comorbidities. Nevertheless, it is reported that higher levels of D-dimer and prolonged PT are associated with a poor prognosis in COVID-19 patients [30, 31, 33, 34]. Such reports are in line with our observations on D-dimer, PT, and INR, all of which are known to be independent risk markers for COVID-19 severity and subsequent fatal outcomes.

Among other inflammatory markers, CRP and ESR are the two most frequently used in some settings, with the former being a direct indicator of the acute phase inflammation and the latter providing an indirect measure. Many studies have found significant correlations between higher CRP levels and complications such as acute lung injury (ALI), acute respiratory disorder stress (ARDS), and mortality in COVID-19 patients [35–37]. Elevated CRP could be due to bacterial coinfection that may occur in severe COVID-19 [38, 39]. Moreover, ESR has been reported as an effective predictor of pneumonia, drug-induced pulmonary toxicity, and coronary heart disease [40–42]. Nonetheless, the relationship between levels of ESR and COVID-19 severity is reported controversially in various studies. While many findings indicate a positive correlation between increased ESR levels and COVID-19 severity in hospitalized patients [43, 44], other studies, including ours, showed no significant relationship [45]. In addition, ferritin, an important inflammatory protein associated with thromboembolic events, ARDS, and COVID-19 severity [37, 46], tended to be higher in severe cases in our study, but probably due to the small number of samples not statistically different.

Nonrespiratory presentations of COVID-19 include laboratory abnormalities in different biochemical parameters. Liver, heart, and renal dysfunctions are among the fatal complications contributing to disease mortality [47]. According to a systematic review [48], the most common laboratory abnormality mentioned in COVID-19 patients was hypoalbuminemia, followed by an increase in AST, ALT, GGT, ALP, and total bilirubin [48, 49], all of which are related to kidney, liver, and bile damage. Our results, however, did not show significant evidence of changes in albumin perhaps, possibly because of the small size of the tested population. Noteworthy, higher AST levels were more prevalent than ALT in severe cases of COVID-19, in line with other reports [48]. Based on the present and previous studies, ALP is at higher levels in COVID-19 patients, in whom the raised LDH could be observed in severe cases where a large number of cells may undergo necrosis due to subclinical tissue damage [26, 50]. The pathogenesis of liver damage in SARS-CoV-2 infection may be due to exacerbation of the underlying liver disease, cytopathic effects caused by the virus, hypoxemia-induced ischemia, drug damage, immune system disorder, and cytokine storm [26, 51, 52].

It is worth mentioning that elevated aminotransferases could be related to myositis rather than liver damage in COVID-19. AST levels can be high along with CK-MB, LDH, and troponin, indicating cardiac problems associated with COVID-19 [49]. In our study, we also report a significant increase in CK-MB levels in severe cases compared to nonsevere patients as well as an increase in creatinine kinase in deceased compared to recovered patients. In addition, acute kidney injury (AKI) and electrolyte disturbances are important complications of hospitalized COVID-19 patients. AKI is thought to occur due to multiple pathophysiological mechanisms, such as multiple organ dysfunction, direct viral entry into the renal tubules, and cytokine release syndrome [53]. BUN is reported in many cases of AKI and was significantly higher in severe cases of COVID-19 [54]. Our findings also suggest that enhanced creatinine levels correlate with a two-fold increased risk of COVID-19 severity and fatal outcomes. Furthermore, findings on the relationship between uric acid levels and disease severity in COVID-19 vary in different studies [55–57]. In this study, we show hyperuricemia in COVID-19 patients and a significant correlation to the outcome of death. Hyperuricemia might be due to direct pathophysiological effects from inflammation, endothelial dysfunction, activation of the renin-angiotensin-aldosterone system, oxidative stress, and insulin resistance, whereas hypouricemia reported in some studies may be due to the effects of cytokine storm on urate transporters [58].

Electrolyte imbalances like hypocalcemia, hypokalemia, and hyponatremia are common manifestations in hospitalized COVID-19 cases. Hypocalcemia is the most common electrolyte disturbance in COVID-19 disease, resulting from viral effects on calcium signaling pathways, vitamin D deficiency, parathyroid imbalance, malnutrition during critical illness, and unsaturated fatty acids in inflammatory responses [29]. In the context of SARS-CoV-2 infection, hypokalemia may result from hyperactivity of the renin-angiotensin-aldosterone system (RAAS), gastrointestinal losses, anorexia due to concurrent illness, or tubular damage caused by ischemia or nephrotoxic agents [59]. Moreover, the occurrence of COVID-19 symptoms, including anorexia, sweating, diarrhea, and vomiting, may lead to excessive sodium excretion and cause hyponatremia. Again, there are contrasting reports on the relationship between electrolyte imbalance and COVID-19 severity. Our study, however, agrees with Martha et al. [60] regarding the association between hypocalcemia and extended hospital and ICU duration in COVID-19, as well as enhanced levels of sodium and potassium positively correlating with disease severity and mortality. This association is true for levels of phosphorus in severe and deceased patients compared to nonsevere and recovered cases. Our OR analysis shows that phosphate levels were higher than reference values accompanied with a higher probability of death, 1.7-fold, in agreement with Hadavi et al. (1.73-fold) [61]. The proven hyperphosphatemia in COVID-19 may be due to cytokines' damaging effects on cells and the subsequent release of phosphate ions [62].

So far, several approaches have been used to predict a poor prognosis in COVID-19 patients, including scoring systems based on physiological factors to assess major organ function [63] and analysis of complex medical datasets based on machine learning studies [64]. The studies showed that scoring systems are not specifically designed for COVID-19, and because of limited clinical data, they may not be applicable for nonsevere patients at the time of admission. Also, there are contradictory results of the SOFA (sequential organ failure assessment) score in predicting the severity of patient mortality [65]. Studies have shown an ability of machine learning methods (logistic regression (LR), support vector machine (SVM), decision tree (DT), random forest (RF), and a deep learning-based method (to improve the accuracy and early screening of COVID-19 diagnosis. The higher AUC of our LR-based predictive model makes it a more conducive method for assisting COVID-19 diagnosis [64]. The findings of this study and previous studies with demographics and existing laboratory measurements are routinely available and useful prognostic markers when the patient is admitted to the hospital. However, risk prediction models for COVID-19 require external validation before widespread clinical use, as severity and prognosis depend on the care of settings and hospital systems.

The current study has some limitations. As a single-center, retrospective study on hospitalized patients, results cannot be generalized to the entire population. The sample size is relatively small, and all 46 laboratory tests were not performed for all patients. This may negatively affect the reproducibility of our data, and we may have underestimated the role of some tests in predicting the severity and mortality. Moreover, information about concomitant diseases is not included in this study due to the lack of access to the patients' personal medical history.

The current study has some limitations. As a single-center, retrospective study on hospitalized patients, its results cannot be generalized to the entire population. The sample size is relatively small, and all 46 tests were not performed for all patients. This issue may negatively affect the reproducibility of our data and have underestimated the role of some tests in predicting the severity and mortality of the disease. Moreover, information about concomitant diseases is not included in this study due to the lack of access to the patients' personal medical history.

## 5. Conclusion

In conclusion, we suggest age, WBC, NLR, PLT, HGB, and INR and age, BUN, and LDH models as potential indicators for severe disease or mortality outcome. Considering the potential of routine hematological and biochemical tests in predicting the course of the COVID-19 disease, we believe that results in various studies using different methods, statistical approaches, population characteristics, and geographical locations, along with our study, may provide the best models for predicting severity. This will improve the careful monitoring of severity predictors and facilitate to enable early clinical intervention for patients, thereby reducing the mortality rate of COVID-19 patients and hopefully helping to control and prevent future epidemics and pandemics.

## Figures and Tables

**Table 1:** Demographic data of all patients: deceased, recovered, severe, and nonsevere COVID-19 cases.

Variables	Total	Outcome		*P* value	Severity		*P* value
		Deceased	Recovered		Severe	Nonsevere	
Number (%)	564 (100)	97 (17.2)	467 (82.8)		104 (18.5)	460 (81.5)	
Sex							
Male (%)	298 (52.8)	50 (16.7)	248 (83.2)		60 (20.2)	238 (79.8)	
Female (%)	266 (47.2)	47 (17.6)	219 (82.4)	0.760	44 (16.6)	222 (83.4)	0.264
Age (Mean ± Std)	55.6 ± 16	66.81 ± 1.6	53.02 ± 0.8	<0.001	66.5 ± 1.7	53.2 ± 0.8	<0.001

Note: data is mean ± SD and median (IQR). *P* values for differences between the two groups were obtained by the Student *t*-test or the Mann–Whitney *U* test. ICU: intensive care unit; IQR: interquartile range.

**Table 2:** Hematological data of all patients: deceased, recovered, severe, and nonsevere COVID-19 cases.

Variables	Normal range	Total	Outcome		*P* value	Severity		*P* value
			Deceased	Recovered		Severe	Nonsevere	
WBC (×10^9^/L)	4.2-10.8	11.7 (8.4-15.8)	11.7 (8.4-15.8)	7.6 (5.3-10.3)	<0.001	11.5 (8.1-15.4)	7.7 (5.3-10.4)	<0.001
LYM (×10^9^/L)	1.5-4.5	0.8 (0.5-1.1)	0.8 (0.5-1.1)	1 (0.6-1.5)	0.007	0.8 (0.5-1.1)	1 (0.7-1.5)	0.002
NEU (×10^9^/L)	1.7-7.9	6 (4-9)	10 (7-14)	6 (4-8)	<0.001	10 (6-14)	8 (6-12)	<0.001
NLR		7 (3.5-11.12)	12.14 (7.5-17.6)	5.6 (3.2-9.8)	<0.001	12.43 (7.3-17.6)	5.6 (3.2-9.8)	<0.001
MO (×10^9^/L)	0.1-0.9	0.1 (0.04-0.17)	0.04 (0-0.13)	0.09 (0-0.16)	0.13	0.06 (0-0.19)	0.1 (0.05-0.16)	0.009
EOS (×10^9^/L)	0.02-0.55	0.09 (0-0.16)	0.04 (0-0.13)	0.09 (0-0.16)	0.03	0.04 (0-0.13)	0.09 (0-0.16)	0.007
RBC (×10^12^/L)	4.2-5.65	4.4 (3.95-4.85)	4.14 (3.5-4.6)	4.4 (4-4.8)	<0.001	4.1 (3.5-4.7)	4.4 (4-4.8)	0.001
HGB (g/dl)	12-15	12.5 ± 2.0	11.9 ± 0.22	12.7 ± 0.09	<0.001	11.8 ± 0.24	12.7 ± 0.09	0.001
PLT (×10^9^/L)	160-385	220 (169-288)	213 (147.5-245)	224 (174-297)	0.002	215 (144.2-251)	223 (174.7-297.2)	0.004
HCT (%)	38-49	38.02 ± 5.2	38.1 ± 0.6	38.4 ± 0.2	0.001	36.5 ± 0.6	38.3 ± 0.2	0.013
MCV (fl)	80-98	87.2 (83.8-90.6)	87 (84.9-90.86)	87 (83.69-90.63)	0.259	87.5 (84.8-90.67)	87.1 (83.1-90.7)	0.385
MCH (pg)	28-32	29.14 (27.5-30.4)	28.9 (27.5-30)	29.2 (27.4-30.5)	0.395	28.9 (27.5-30.1)	29.2 (27.5-30.5)	0.504
MCHC (g/dl)	33-36	33.0 ± 1.5	32.6 ± 0.15	33.1 ± 0.07	0.003	32.6 ± 0.15	33.1 ± 0.07	0.001
ESR (mm/hr)	0-20	46 (27-66)	54 (34-81)	44 (26-64)	0.025	50.5 (30-81.5)	45 (26.75-65)	0.249
INR	0.8-1.2	1.1 (1-1.2)	1.2 (1.1-1.45)	1.08 (1-1.17)	<0.001	1.23 (1.1-1.48)	1.08 (1-1.15)	<0.001
PT (sec)	11-13	13.2 (12.5-14.1)	13.95 (13.1-16)	13 (12.5-13.8)	<0.001	14.2 (13.1-16.17)	13 (12.5-13.8)	<0.001
PTT (sec)	21-32	32 (28.1-36.1)	33 (28.9-37.7)	31.7 (28-35.1)	0.063	32.6 (28.8-37)	31.8 (28-35.2)	0.282

Note: Data are mean ± SD and median (IQR). *P* values for differences between the two groups were obtained by the Student *t*-test or the Mann–Whitney *U* test. IQR: interquartile range; WBC: white blood cells; LYM: lymphocytes; NEU: neutrophil; NLR: neutrophil-to-lymphocyte ratio; MO: monocyte; monocyte-to-lymphocyte ratio; EOS: eosinophil; RBC: red blood cells; HGB: hemoglobin; PLT: platelet cells; HCT: hematocrit; MCV: mean corpuscular volume; MCH: mean corpuscular hemoglobin; MCHC: mean corpuscular hemoglobin concentration; ESR: erythrocyte sedimentation rate; INR: international normalized ratio; PT: prothrombin time; PTT: partial thromboplastin time. The threshold for significance is the Bonferroni-corrected from *P* < 0.05 to *P* < 0.001.

**Table 3:** Biochemical data of all patients: deceased, recovered, severe, and nonsevere COVID-19 cases.

Variables	Normal range	Total	Outcome		*P* value	Severity		*P* value
			Deceased	Recovered		Severe	Nonsevere	
BUN (mg/dl)	8-20	20 (30-14)	37 (22-56.5)	18 (14-26)	<0.001	34 (22-55)	18 (14-27)	<0.001
Creatinine (mg/dl)	0.7-1.3	1.1 (0.9-1.3)	1.3 (1-2.3)	1 (0.9-1.2)	<0.001	1.3 (1-3.8)	1 (0.9-1.2)	<0.001
Na (mEq/L)	136-145	141 (137-144)	143 (138-145)	141 (137-144)	0.002	143 (138-145)	141 (137-144)	0.02
K (mEq/L)	3.5-5	4.1 (3.8-4.5)	4.3 (3.8-4.9)	4.1 (3.8-4.5)	0.045	4.3 (3.7-4.9)	4.1 (3.8-4.5)	0.04
Ca (mg/dl)	8.6-10.2	8.8 (8.5-9.3)	8.7 (8.4-9)	8.9 (8.5-9.3)	0.01	8.65 (8.4-9)	8.9 (8.6-9.3)	0.008
Mg (mEq/L)	1.6-2.6	2 (1.81-2.21)	1.98 (1.8-2.23)	2 (1.8-2.2)	0.878	2.03 (1.8-2.2)	1.99 (1.81-2.2)	0.373
P (mg/dl)	3.0-4.5	3.6 (3.2-4.2)	4.1 (3.3-4.9)	3.6 (3.2-4.1)	0.008	4.1 (3.3-5.1)	3.6 (3.2-4.1)	0.018
LDH (U/L)	80-225	646 (502-828)	736 (626.5-995.5)	598 (471-763)	<0.001	741 (553-10496)	607 (483 -757)	<0.001
AST (U/L)	10-40	44 (31-68)	50 (36-72)	42 (31-67)	0.049	49.5 (33-73)	42.5 (31-67)	0.107
ALT (U/L)	10-40	36.5 (22-62.25)	33.5 (18.25-52)	36.5 (22-66)	0.429	32 (18-52)	37 (22. -65.75)	0.478
ALP (U/L)	30-120	184 (148-242)	227 (160-314.75)	180 (145-229)	<0.001	230 (159-318)	180 (146-227)	<0.001
Amylase (U/L)	25-125	53.8 ± 44.8	74.5 ± 24.7	47.6 ± 4	0.309	74.0 ± 22.3	46.6 ± 4	0.511
CPK (U/L)	20-200	119 (60-244)	166.5 (78.75-406)	114 (57-238.25)	0.035	160 (74.5-390)	118 (58-239)	0.09
CK-MB (U/L)	5-25	16 (12-25.12)	20 (15-30)	16 (11.25-23.5)	0.065	23 (16-32.12)	15.5 (11-22)	0.008
TG (mg/dl)	<150	112.8 ± 48	97.6 ± 8.7	114.3 ± 9.9	0.487	95.0 ± 8.4	114.8 ± 9.9	0.118
Cholesterol (mg/dl)	<200	141.2 ± 33	124.4 ± 11.0	144.0 ± 6.7	0.236	120.0 ± 8.9	144.8 ± 6.7	0.118
FBS (mg/dl)	70-99	167 (115.5-248.5)	184 (121-258)	154 (110-231)	0.157	172 (117-258)	159 (113.5-243)	0.548
BS (mg/dl)	70-120	145 (108-236)	139 (109-211)	148 (109-249)	0.385	140 (109-222)	148 (109-252)	0.521
HbA1C (%)	4.0-5.6	7.5 ± 1.41	7.3 ± 0.24	7.7 ± 0.19	0.283	7.09 ± 0.22	7.8 ± 0.19	0.071
D-dimer (*μ*g/dL)	<0.35	0.52 (0.29-1.14)	0.75 (0.53-4.9)	0.46 (0.23-1.1)	0.0.009	0.94 (0.53-4.9)	0.46 (0.25-1.1)	0.016
D-bilirubin (mg/dl)	0.1-0.3	0.2 (0.1-0.3)	0.3 (0.2-0.4)	0.2 (0.1-0.3)	0.002	0.3 (0.2-0.4)	0.2 (0.1-0.3)	0.001
T-bilirubin (mg/dl)	0.3-1.0	0.7 (0.5-1)	0.8 (0.6-1.2)	0.7 (0.5-1)	0.013	0.8 (0.6-1.2)	0.7 (0.5-1)	0.004
CRP (mg/dl)	0-50	36 (16.07-57.4)	40.2 (25.4-64.4)	33 (14.5-50)	0.008	39.95 (25.55-64.1)	33 (14.5-51)	0.02
Iron (*μ*g/dL)	50-150	42 (37.5-90.5)	40.5 (37.5-204.75)	49 (25-103.5)	0.864	40 (33-73)	55 (40-110)	0.306
Ferritin (ng/mL)	24-336	702.5 (349.6-800)	728.3 (425.3-800)	657 (337.1-800)	0.23	734 (556.62-800)	615 (326.75-800)	0.052
TIBC (*μ*g/dL)	250-310	222 ± 55	186 ± 40	235 ± 16	0.198	194 ± 30	237 ± 19	0.227
UA (mg/dl)	3.0-7.0	6.2 ± 3.6	9.4 ± 1.7	5.4 ± 0.9	0.049	8.8 ± 2.3	5.9 ± 1.0	0.181
Total protein (g/dL)	5.7-8.2	6.04 ± 1.05	5.85 ± 0.3	6.2 ± 0.2	0.346	5.85 ± 0.3	6.2 ± 0.2	0.346
Albumin (g/dL)	3.2-4.8	3.7 (3.2-3.9)	3.4 (3.05-3.65)	3.8 (3.3-4)	0.001	3.4 (3-3.7)	3.7 (3.2-3.9)	0.001

Note: data are mean ± SD and median (IQR). *P* values for differences between the two groups were obtained by the Student *t*-test or the Mann–Whitney *U* test. IQR: interquartile range; BUN: blood urea nitrogen; Cr: creatinine; Na: sodium; K: potassium; Ca: calcium; Mg: magnesium; P: phosphorus; LDH: lactate dehydrogenase; AST: aspartate aminotransferase; ALT: alanine transaminase; ALP: alkaline phosphatase; CPK: creatine phosphokinase; CK-MB: creatine kinase-MB; TG: triglyceride; FBS: fasting blood sugar; BS: blood sugar; HbA1C: hemoglobin A1C; CRP: C-reactive protein; TIBC: total iron-binding capacity; UA: uric acid. The threshold for significance is the Bonferroni-corrected from *P* < 0.05 to *P* < 0.001.

**Table 4:** Logistic regression analysis of the models of predictors for severity and mortality.

Parameters	Multivariate model for mortality		Parameters	Multivariate model for severity	
	OR (95% CI)	*P* value		OR (95% CI)	*P* value
WBC	1.13 (1.08-1.1)	<0.001	WBC	1.1 (1.05-1.15)	<0.001
NLR	1.10 (1.05-1.14)	<0.001	NLR	1.1 (1.0-1.14)	<0.001
EOS	0.23 (0.01-3.03)	0.26	EOS	0.35 (0.02-3.8)	0.35
PLT	0.996 (0.994-1)	0.02	PLT	0.997 (0.994-1)	0.02
RBC	0.66 (0.45-0.96)	0.03	RBC	0.6 (0.4-0.9)	0.03
HGB	0.82 (0.72-0.93)	0.002	HGB	0.8 (0.7-0.9)	0.002
HCT	0.94 (0.89-0.99)	0.019	HCT	0.94 (0.89-0.99)	0.02
ESR	1.01 (1.04-1.09)	0.037	MO	0.76 (0.1-5.4)	0.78
MCHC	0.75 (0.63-0.89)	<0.001	MCHC	0.7 (0.6-0.9)	0.001
INR	8.48 (2.42-29.6)	<0.001	INR	8 (2-29)	0.001
PT	1.21 (1.07-1.37)	0.002	PT	1.2 (1.0-1.3)	0.002
BUN	1.05 (1.03-1.06)	<0.001	BUN	1.04 (1.01-1.06)	<0.001
Creatinine	2.28 (1.61-3.22)	<0.001	Creatinine	2.2 (1.5-3.1)	<0.001
Na	1.09 (1.04-1.15)	<0.001	Na	1.05 (0.99-1.1)	0.056
K	1.57 (1.07-2.31)	0.01	K	1.5 (1.0-2.2)	0.03
Ca	0.55 (0.30-1.03)	0.06	Ca	0.5 (0.2-1)	0.06
P	1.7 (1.22-2.36)	0.001	P	1.8 (1.2-2.5)	0.001
LDH	1.002 (1.001-1.003)	<0.001	LDH	1.002 (1.001-1.003)	<0.001
AST	1 (0.99-1.005)	0.95			
ALP	1.003 (1.001-1.005)	0.004	ALP	1.003 (1.001-1.006)	0.001
CPK	1 (0.99-1)	0.35	CKMB	0.99 (0.97-1.00)	0.9
D-dimer	1.31 (1.001-1.7)	0.049	D-dimer	1.3 (1.0-1.7)	0.03
D-bilirubin	1.52 (0.86-2.6)	0.14	D-bilirubin	1.5 (0.8-2.7)	0.1
T-bilirubin	1.04 (0.8-1.3)	0.68	T-bilirubin	1.0 (0.8-1.3)	0.49
UA	1.5 (0.9-2.5)	0.09			
CRP	1/01 (1.00-1.02)	0.01	CRP	1.0 (0.9-1.01)	0.16
Albumin	0.2 (0.1-0.6)	0.002	Albumin	0.2 (0.1-0.5)	0.002

Note: data is adjusted with age.

**Table 5:** ROC analysis of laboratory parameters.

	*P* value from Hosmer-Lemeshow test	Accuracy	AUC (95% CI)	*P* value from predicted probability
Predictors for mortality				
“WBC; age”	0.171	81.2	0.80 (0.74-0.85)	<0.001
“PLT; age”	0.732	81.1	0.75 (0.69-0.80)	<0.001
“NLR; age”	0.870	81.3	0.80 (0.75-0.85)	<0.001
“RBC; age”	0.334	81.2	0.75 (0.69-0.81)	<0.001
“HGB; age”	0.755	81.2	0.76 (0.70-0.81)	<0.001
“HCT; age”	0.855	81.2	0.75 (0.69-0.80)	<0.001
“ESR; age”	0.706	81.1	0.77 (0.70-0.83)	<0.001
“MCHC; age”	0.951	81.1	0.75 (0.70-0.81)	<0.001
“INR; age”	0.223	78.1	0.77 (0.72-0.83)	<0.001
“PT; age”	0.333	77.8	0.77 (0.71-0.82)	<0.001
Model 1: “age, WBC, NLR, PLT, HGB, and INR”	0.367	82.7	0.872 (0.83-0.91)	<0.001
“BUN; age”	0.632	81	0.80 (0.75-0.86)	<0.001
“Creatinine; age”	0.418	81	0.78 (0.72-0.83)	<0.001
“Na; age”	0.471	80.9	0.75 (0.69-0.81)	<0.001
“K; age”	0.382	80	0.74 (0.68-0.80)	<0.001
“P; age”	0.754	75	0.77 (0.69-0.85)	<0.001
“LDH; age”	0.559	78.1	0.83 (0.78-0.88)	<0.001
“ALP; age”	0.743	77.8	0.76 (0.69-0.82)	<0.001
“D-dimer; age”	0.226	77.4	0.75 (0.60-0.86)	<0.001
“CRP; age”	0.022	80.8	0.75 (0.69-0.81)	<0.001
“Albumin; age”	0.341	67.3	0.73 (0.64-0.83)	<0.001
Model 2: “age, BUN, and LDH”	0.248	81	0.85 (0.80-0.91)	<0.001

Predictors for severity				<0.001
“WBC; age”	0.258	82.3	0.77 (0.71-0.82)	<0.001
“NLR; age”	0.983	82.4	0.78 (0.72-0.83)	<0.001
“PLT; age”	0.753	82.5	0.75 (0.69-0.81)	<0.001
“RBC; age”	0.413	82.3	0.74 (0.68-0.80)	<0.001
“HGB; age”	0.557	82.3	0.75 (0.69-0.81)	<0.001
“HCT; age”	0.803	82.3	0.73 (0.67-0.79)	<0.001
“MCHC; age”	0.695	82.5	0.75 (0.69-0.81)	<0.001
“INR; age”	0.231	80.1		Nonvalid
“PT; age”	0.748	79.7		Nonvalid
Model 3: “age, WBC, NLR, PLT, HGB, and INR”	0.576	84.7	0.86 (0.81-0.90)	<0.001
“BUN; age”	0.669	82.4	0.80 (0.74-0.86)	<0.001
“Creatinine; age”	0.279	82.4	0.77 (0.71-0.83)	<0.001
“K; age”	0.656	82.5	0.73 (0.67-0.79)	<0.001
“P; age”	0.959	75.5	0.79 (0.71-0.86)	<0.001
“LDH; age”	0.739	79.1	0.80 (0.74-0.86)	<0.001
“ALP; age”	0.874	79.4	0.78 (0.71-0.84)	<0.001
“D-dimer; age”	0.541	79	0.77 (0.55-0.85)	<0.001
“Albumin; age”	0.211	67.3	0.75 (0.65-0.84)	<0.001
Model 4: “age, BUN, and LDH”	0385	81.7	0.85 (0.80-0.91)	<0.001

## Data Availability

The datasets during and/or analyzed during the current study are available from the corresponding author on reasonable request.

## Abbreviations

SARS-CoV-2: Severe acute respiratory syndrome coronavirus-2
COVID-19: Coronavirus disease
rRT-PCR: Real-time reverse transcription-polymerase chain reaction
CRP: C-reactive protein
LDH: Lactate dehydrogenase
IQR: Interquartile range
ROC: Receiver operating characteristic
NLR: Neutrophil to lymphocyte ratio
ALI: Acute lung injury
Acute ARDS: Respiratory disorder stress
AKI: Acute kidney injury
LR: Logistic regression
SVM: Support vector machine
DT: Decision tree
RF: Random forest
WBC: White blood cells
LYM: Lymphocytes
NEU: Neutrophil
MO: Monocyte
EOS: Eosinophil
RBC: Red blood cells
HGB: Hemoglobin
PLT: Platelet cells
HCT: Hematocrit
MCV: Mean corpuscular volume
MCH: Mean corpuscular hemoglobin
MCHC: Mean corpuscular hemoglobin concentration
ESR: Erythrocyte sedimentation rate
INR: International normalized ratio
PT: Prothrombin time
PTT: Partial thromboplastin time
BUN: Blood urea nitrogen
Cr: Creatinine
Na: Sodium
K: Potassium
Ca: Calcium
Mg: Magnesium
P: Phosphorus
LDH: Lactate dehydrogenase
AST: Aspartate aminotransferase
ALT: Alanine transaminase
ALP: Alkaline phosphatase
CPK: Creatine phosphokinase
CK-MB: Creatine kinase-MB
TG: Triglyceride
FBS: Fasting blood sugar
BS: Blood sugar
HbA1C: Hemoglobin A1C
TIBC: Total iron-binding capacity
UA: Uric acid.
